# Recent Progress and Prospect in Studying Selective Inhibitors Toward Bromodomain Family Members

**DOI:** 10.3390/molecules31050837

**Published:** 2026-03-02

**Authors:** Jianzhong Chen, Yu’e Huang, Jian Wang, Wanchun Yang

**Affiliations:** 1 School of Science, Shandong Jiaotong University, Jinan 250357, China; wangjian_lxy@sdjtu.edu.cn; 2 Jinan Foreign Language School, Jinan 250108, China; yingling_1979@126.com

**Keywords:** bromodomain, selective inhibitors, gatekeeper residue, deep learning

## Abstract

Bromodomain (BRD)-containing proteins are gaining attention as key targets in epigenetic drug development. BRDs bind to acetylated lysine residues on histones and other proteins, significantly impacting transcriptional regulation and chromatin remodeling. As our grasp of bromodomain structures and biochemistry deepens, the momentum behind developing small-molecule inhibitors for these BRD domains is triggered and potent inhibitors targeting different family members of BRDs are proposed. In addition, computational simulations have also played a significant role in advancing inhibitor design for the BRD family. This review delves into recent breakthroughs in small-molecule BRD receptor inhibitors and computational studies, spotlighting their biological impact and therapeutic potential, and outlining the research road ahead. This review is expected to provide guidance for future drug design of BRD inhibitors.

## 1. Introduction

Bromodomains (BRDs), protein modules of around 110 amino acids, specifically bind to acetylated lysine residues on histones and other proteins [1,2]. To date, 46 human proteins containing BRD have been identified, with many playing roles in transcriptional regulation and chromatin remodeling. The bromodomain and extraterminal domain (BET) proteins, including BRD2, BRD3, BRD4, and BRDT, represent a significant subgroup of the BRD family. BET proteins are crucial in chromatin biology, influencing transcriptional processes linked to various disease states [3]. Given their involvement in cancer and inflammation [4,5,6,7,8], the BRD family has emerged as promising targets for gene transcription and inhibiting therapies [9,10,11]. Notably, BET protein inhibitors have exhibited varying degrees of effectiveness across a range of conditions, including solid and hematological malignancies, cardiovascular diseases, autoimmune disorders, and inflammatory conditions.

**Figure 1:**
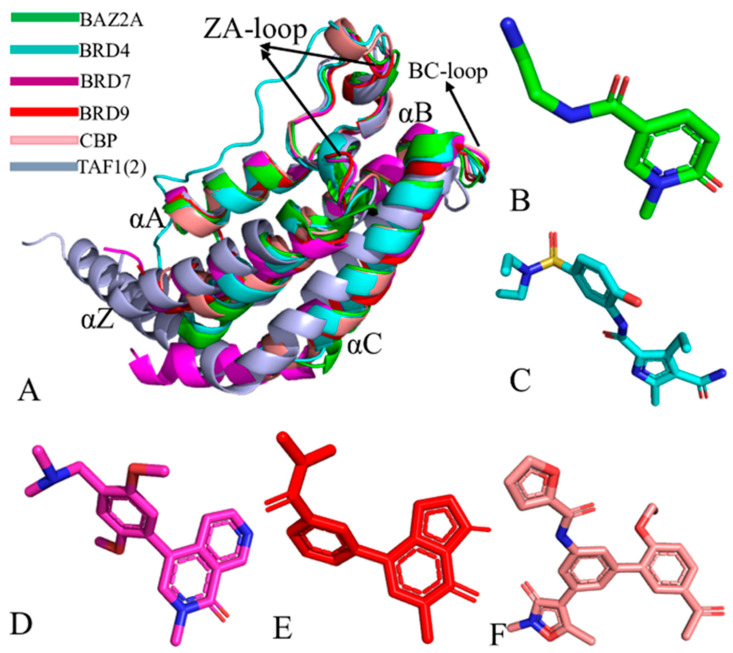
Molecular structures: (**A**) alignments of several representative BRD family members, including BAZ2A (PDB: 6FG6), BRD4 (PDB: 5D26), BRD7 (PDB: 6V1F), BRD9 (PDB: 5I7X), CBP (PDB: 6FQO), and TAF1(2) (PDB: 5I29), shown in cartoon modes; (**B**) BAZ2A inhibitor D8Q; (**C**) BRD4 inhibitor L28; (**D**) BRD7 inhibitor 5U6; (**E**) BRD9 inhibitor 67B; and (**F**) CBP inhibitor E2T. In this figure, the crystal structures are from protein data bank. In this figure, the blue indicates the nitrogen atom while the yellow indicates the sulfur atom.

In addition to BET proteins such as BRD2, BRD3, BRD4, and BRDT, various homologous BRD proteins have been identified [12,13,14]. For example, BAZ2A and BAZ2B, which are located near zinc finger domain protein 2A and 2B, share 57% sequence identity and have a unique binding pocket that accommodates acetyl-lysine [15,16,17,18,19,20]. CREB binding protein (CBP) associates with chromatin via its bromodomain and can modify chromatin and recruit transcription proteins to regulate gene expression by means of its histone acetyltransferase activity [21,22,23,24,25]. The second bromodomain of human transcription initiation factor TFIID subunit 1 (TAF1(2)) is overexpressed in patients of many cancers and plays a key role in acute myeloid leukemia 1 (AML1)-ETO fusion gene expression [26,27,28,29,30]. Bromodomain-containing protein 9 (BRD9) exhibits diverse physiological functions in multiple tissues and is a core subunit of SWI/SNF complexes, contributing to leukemia growth [31,32,33,34,35]. Moreover, BRD7 [34,36], a paralog of BRD9, is a subunit of poly-bromo-associated BAF SWI/SNF complexes. These BRD family members share a similar structural topology (Figure 1A), including four alpha-helices (αZ, αA, αB, and αC) and two flexible loop regions (ZA-loop and BC-loop), with the ZA-loop and BC-loop playing crucial roles in inhibitor binding. Furthermore, inhibitors of BAZ2A, BRD4, BRD7, BRD9, and CBP, corresponding to D8Q [37], L28 [38], 5U6 [34], 67B [26], and E2T [22] (Figure 1B–F), have different binding selectivity toward different BRD family members.

Given the crucial roles that BRD family members play in a variety of human diseases, the development of small-molecule BRD receptor inhibitors has garnered significant attention within the research community. First-generation BET inhibitors, including compounds such as JQ1 [39,40] and I-BET151 [41,42,43,44], functioned as pan-BET inhibitors, targeting both BRD BD1 and BRD BD2 [13]. Notably, selective inhibitors toward BRD BD1 and BRD BD2 have been provided to treat various cancers, such as prostate cancer, etc. [45,46,47,48]. Crystallographic studies demonstrated that JQ1 could bind to BRD4, BRD2, and BRDT, albeit with varying degrees of inhibitory efficacy. These compounds exhibited robust anti-proliferative effects across multiple cancer cell lines, offering valuable insights into the pathophysiological roles of BET proteins. However, their limited selectivity across BRD family members has restricted their utility in precisely delineating the specific contributions of individual domains. Consequently, the development of clinically available BRD inhibitors with high selectivity for distinct BRD family remains a formidable challenge for future research. Several BET BRD inhibitors, such as CPI-0610 [49,50,51], I-BET762 [52,53,54], and RVX-208 [55,56], have advanced to clinical-trial stages, reflecting the ongoing efforts in this field. The inhibitor GSK2801 [18,57,58] exhibits a degree of selectivity for BAZ2A and BAZ2B and has been employed to modulate the transcription of non-coding RNA. Meanwhile, LP99 [59], which demonstrates improved selectivity and anti-proliferative activity in cellular models, has been utilized to effectively inhibit the activities of BRD7 and BRD9. In summary, the pursuit of highly selective BRD inhibitors continues to be an active area of investigation, with researchers striving to overcome existing limitations and advance our understanding of BRD biology in disease contexts.

In addition to experimental approaches, computational simulation technologies, such as molecular docking, molecular dynamics (MD) simulations [60,61,62,63,64,65], QM/MM calculations [66], Markov state models (MSMs) [67,68,69], and binding free energy calculations [62,70,71,72,73,74], have become instrumental in understanding the interaction mechanisms between ligands and their targets. These computational methods have been effectively employed to elucidate the molecular mechanisms governing binding and selectivity within the BRD family [66,75,76,77]. More recently, the integration of machine-learning and deep-learning techniques has further advanced this field by identifying key hotspots involved in ligand–target interactions [78,79,80,81,82,83,84]. Significantly, these technologies have also been utilized to investigate the free energy profiles of BRD members, facilitate the discovery of novel BRD inhibitors, and enhance our comprehension of their binding mechanisms [85,86,87,88,89]. Drawing from these advancements, it is evident that AI technologies hold great promise for the development of clinically available drugs targeting BRD family members.

## 2. Selective Inhibitors of BRD Family Members

### 2.1. Selective Inhibitors Toward BRD2, BRD3, BRD4, and BRDT

The BRD and BET family comprises four members: BRD2, BRD3, BRD4, and BRDT. These BET proteins are characterized by the presence of two structurally similar proteins, BRD BD1 and BRD BD2, which have the ability to bind to acetyl-lysine residues located on the histone tails of chromatin. Consequently, BRD-containing proteins function as epigenetic “readers”. They bind to acetyl-lysine through their bromodomains, thereby regulating gene expression. Researchers have reported numerous BRD4 inhibitors, several of which are currently undergoing clinical trials for cancer therapy. JQ1 [15,40], the first potent BRD4 inhibitor with a triazolothienodiazepine skeleton, has been extensively utilized as a probe. It has been instrumental in exploring the biological functions of BET proteins in relation to the treatment of various human diseases.

**Figure 2:**
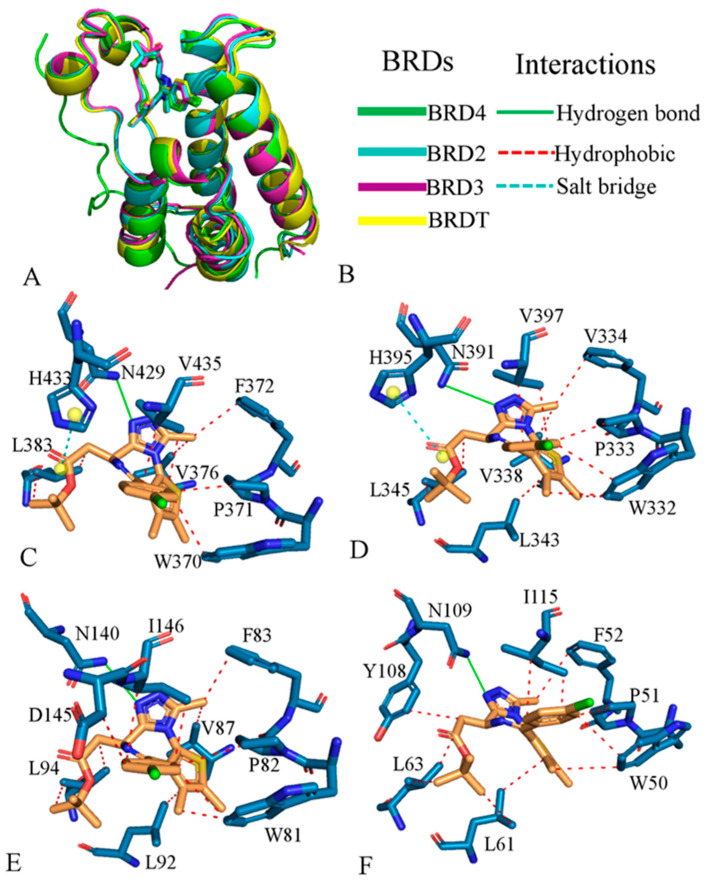
Binding of JQ1 to BRD2, BRD3, BRD4, and BRDT: (**A**) structural alignment of JQ1-bound BRD2, BRD3, BRD4, and BRDT, which respectively correspond to PDB IDs 3ONI, 3S92, 3MXF, and 4FLP; (**B**) types of BRDs and interactions; (**C**) interactions of JQ1 with key residues in BRD2; (**D**) interactions of JQ1 with key residues in BRD3; (**E**) interactions of JQ1 with key residues in BRD4; and (**F**) interactions of JQ1 with key residues in BRDT. In this figure, the crystal structures are from protein data bank. The blue atoms indicate nitrogen atoms and the orange represents the ligand JQ1. The red dotted lines describe hydrophobic interactions, the cyan lines indicate the CH-π interactions and the green solid lines represent hydrogen bonding interactions.

Recent studies have shown that JQ1 binds competitively to the acetyl-lysine recognition motifs of BRD members, including BRD2, BRD3, BRD4, and BRDT, which display minor structural differences (Figure 2A). The high potency and specificity of JQ1 toward these BRD proteins can be attributed to the co-crystal structures (PDB IDs: 3ONI [40], 3S92 [https://doi.org/10.2210/pdb3S92/pdb, accessed on 11 December 2025], 3MXF [40], and 4FLP [90]) with BET family members. These structures reveal excellent shape complementarity with the acetyl-lysine binding cavity and key residues, exhibiting similar interaction patterns (Figure 2B). In the JQ1-BRD2 complex, residues W370, P371, F372, V376, L383, N429, H433, and V435 play significant roles in binding (Figure 2C), as identified by the protein–ligand interaction profiler (PLIP) [91]. For BRD3, residues W332, P333, F334, V338, L343, L345, N391, H395, and V397 form key interactions with JQ1 (Figure 2D). In the JQ1-BRD4 complex, W81, P82, F83, V87, L92, L94, N140, D145, and I146 contribute significantly to JQ1 binding (Figure 2E). As for JQ1-BRDT binding, residues W50, P51, F52, L61, L63, Y108, N109, and I115 are responsible for the main forces (Figure 2F). By now, selective inhibitors toward BRD BD1 and BRD BD2 have been designed to treat various cancers, such as prostate cancer; inflammation; and autoimmune disease [45,46,47,48]. In addition, different groups also paid efforts to develop potent selective compounds on BRD2 and BRD4 [92,93,94,95,96]. Among the four BRD family members, the Trp-Pro-Phe motif and Asn share conserved interactions with JQ1. The aforementioned residues have proven to be effective targets for potent BRD inhibitors.

Toward these target sites, Li et al. identified BRD BD2-selective inhibitors XY153 and XY221, showing efficiency in inhibition of the BRD BD2 [97]. Cui et al. optimized JQ1 structures to obtain lead compounds 26 and 30, which demonstrated 15 and 18 nM affinity for BRD BD1 and over 500-fold selectivity against BRD BD1 and BRD BD2 [98]. Davison et al. used FragLites and PepLites to screen compounds and map ligand interactions of BRD4 and ATAD2 [99]. Overall, significant progress in BRD inhibitor development has been achieved in more works [97,100].

### 2.2. Selective Inhibitors of BRD9 and BRD7

BRD7 and BRD9, two proteins with BRD domains, can bind to acetylated lysines on histone H3 [15]. Despite their single BRD domains being structurally similar (62% sequence identity), full-length BRD7 and BRD9 have quite different cellular functions [101]. BRD9 is part of noncanonical BRG1/BRM-associated factor (ncBAF), a BAF complex subtype, whereas BRD7 is in the polybromo-associated BAF (PBAF) complex. BRD9 is a focus in mSWI/SNF chromatin-remodeling complexes as a drug target. However, BRD7′s role in human biology is not fully understood. Nonselective BRD9 inhibitors may inhibit BRD7 off-target, causing unwanted pleiotropic effects [102]. Therefore, designing highly selective BRD9 inhibitors over BRD7 is crucial for cancer treatment.

Recent efforts have concentrated on creating selective BRD9 inhibitors relative to BRD7. Karim et al. examined various inhibitors biochemically and structurally [34]. They tested known BRD7/9 inhibitors, such as BI7273, BI9564, I-BRD9, TP-472, and bromosporine. Also studied were newly found dual kinase-BRD7/9 inhibitors TG003, Sunitinib, and PF-477736. These showed differing BRD9 potency and selectivity over BRD7. Notably, TG003, Sunitinib, and PF-477736 demonstrated high selectivity. TG003 inhibits Clk1 and Clk4, with IC50 values of 20 and 15 nM, respectively, about 250 times that of BRD7. Sunitinib affects multiple tyrosine kinases, with IC50 values below 10 nM, roughly 1000 times that of BRD7. PF-477736 inhibits Chk1 with a Ki of 0.49 nM, approximately 20,000 times that of BRD7 [34]. Theodoulou and coauthors used I-BRD9 as a cell-active chemical probe to design amide 17, showing over 700-fold selectivity beyond the BET family and more than 70-fold against 34 tested bromodomains [31]. Zoppi et al. found VZ185, a potent, rapid, and selective BRD9 degrader, to also be effective against the closely related BRD7 [103]. The groups of Dykhuizen designed and identified two inhibitors, 1-78 and 2-77, with increased selectivity toward BRD7 and binding ability of submicromolar to BRD7. Their results suggested that these two inhibitors keep key interactions with the asparagine and tyrosine residues critical for acetylated lysine binding [104]. In summary, selective inhibitors of BRD7 over BRD9 and other BRD inhibitors have advanced significantly [33,105].

### 2.3. Selective Inhibitors of BAZ2A and BAZ2B

BAZ2A and BAZ2B, full name bromodomain adjacent to zinc finger domain proteins 2A and 2B, are integral to distinct ISWI chromatin-remodeling complexes. BAZ2A is part of the nucleolar remodeling complex (NoRC), while BAZ2B is in the BAZ2B-containing remodeling factor (BRF) [106]. They play crucial roles in chromatin remodeling and non-coding RNA regulation, impacting gene expression, chromatin modification, cell differentiation, and metabolism. Their BRD domains bind to acetylated histones, steering the NoRC complex or chromatin remodelers to particular chromatin sites, thereby influencing gene transcription and chromatin structure. BAZ2A and BAZ2B have been significant targets in drug design. However, their shallow pocket makes them among the least druggable bromodomains [107]. Despite this, selective inhibitors targeting these two proteins have been proposed [107,108].

**Figure 3:**
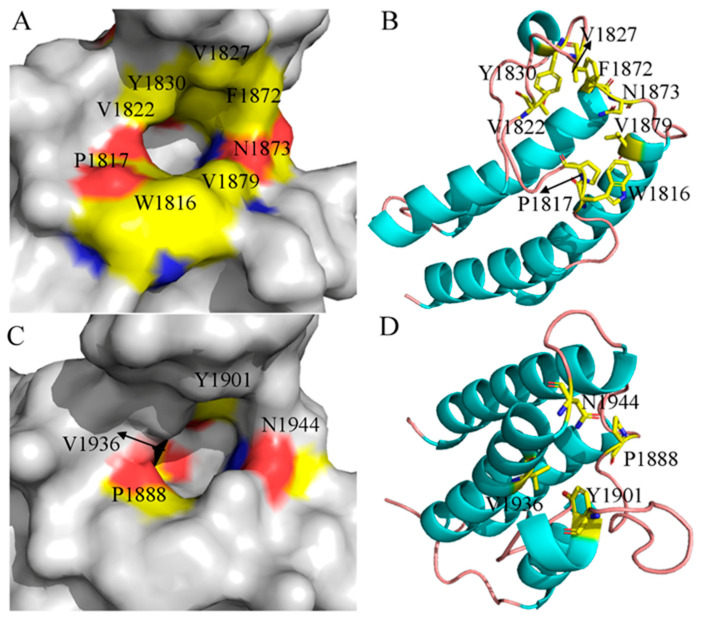
Binding pocket and hotspots of BAZ2A and BAZ2B: (**A**) binding pocket of BAZ2A, (**B**) key residues of BAZ2A, (**C**) binding pocket of BAZ2B and key residues of BAZ2B and (**D**) key residues of BAZ2B. In this figure, the pocket is shown in surface modes, while key residues are indicated in stick modes. In surface modes, the yellow describes carbon atoms of residues, the red indicates oxygen atoms, and the blue reflects the nitrogen atoms.

The crystal structure of BAZ2A (PDB ID: 5MGJ) [107] displays a shallow binding pocket (Figure 3A). The target sites are primarily made up of residues W1816, P1817, V1822, V1827, Y1830, F1872, N1873, and V1879, which are located at the ZA-loop, BC-loop, and αC of BAZ2A (Figure 3B). Furthermore, the structure of the inhibitor–BAZ2B complex (PDB ID: 5E9K) [108] unveils a shallow pocket of BAZ2B. This pocket is shaped by four key residues, namely P1888, Y1901, V1936, and N1944, from the ZA-loop, BC-loop, and αC of BAZ2B (Figure 3C,D). Based on these pockets and target sites, a number of selective inhibitors have been designed to target BAZ2A over BAZ2B. Spiliotopoulos et al. solved the crystal structures of the complex of ligands 1, 2, and 3 with the BAZ2B bromodomain, which mainly differs from BAZ2A in the gatekeeper residue (Val1879 in BAZ2A and Ile1950 in BAZ2B) [107]. Lolli et al. carried out high-throughput fragment docking into the BAZ2B bromodomain and evaluated the binding modes of several fragments to BAZ2B [108]. Additionally, the work of Cazzanelli et al. revealed that TP-238, GSK4027, and UP39 offer additional head group and unique binding modes for the development of new BAZ2 inhibitors [19]. These advancements, along with some other works, provide valuable insights for designing selective inhibitors that target BAZ2A over BAZ2B [18,109,110].

### 2.4. Gatekeeper Residues and Design of BRD Inhibitors

**Figure 4:**
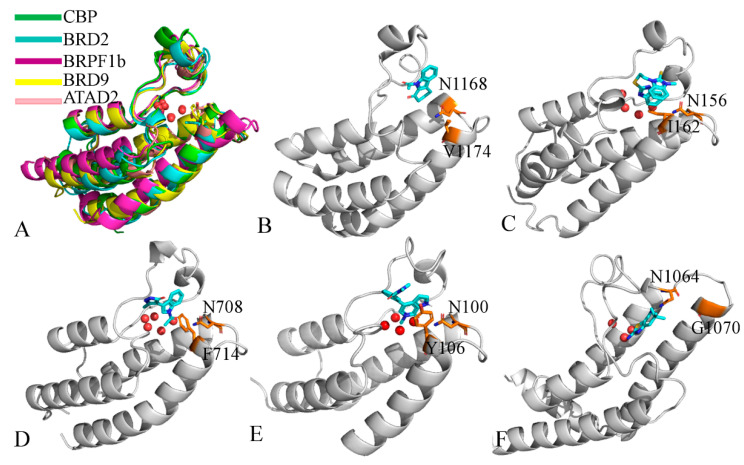
Gatekeeper residues in different BRDs and conserved water molecules: (**A**) structural superimpositions of different BRDs, (**B**) gatekeeper residue V1174 in CBP (PDB ID: 2D82), (**C**) gatekeeper residue I162 in BRD2 (PDB ID: 3AQA), (**D**) gatekeeper residue F714 in BRPF1B (PDB ID: 5D7X), (**E**) gatekeeper residue Y106 in BRD9 (PDB ID: 5I7X), and (**F**) gatekeeper residue G1070 in ATAD2 (PDB ID: 4TZ8). In this figure, water molecules are shown in red ball modes. The blues indicate nitrogen atoms and the oranges represent key gatekeeper residues.

The acetyl-lysine region features a conserved asparagine amino acid crucial for recognizing acetyl-lysine histone peptides. The ZA and BC channels are individually formed by residues from their respective loops, namely a hydrophobic “shelf” area and the first residue of the αC helix, termed as the gatekeeper residue, typically hydrophobic. PDB structural studies show that the size of the gatekeeper residue varies across bromodomains, mainly encompassing glycine, valine, isoleucine, tyrosine, and phenylalanine. Figure 4A illustrates the structural superimpositions of CBP, BRD2, BRPF1B, BRD9, and ATAD2, highlighting gatekeeper residues and crystal water molecules in the binding pocket. Their gatekeeper residues are V1174 (CBP from PDB 2D82) [111], I162 (BRD2 from 3AQA) [112], F174 (BRPF1B from 5D7X) [113], Y106 (BRD9 from 5I7X) [26], and G1070 (ATAD2 from 4TZ8) [114], which are depicted in Figure 4B–F. Based on the difference among BRD members, it is vital to consider the variations in shelf residues and the gatekeeper residue for design of selective inhibitors, as these can significantly impact binding affinity of inhibitors to BRD members.

In light of the effect of gatekeeper residues, several studies have delved into this matter. For example, Meslamani and colleagues demonstrated that hydrophobic gatekeeper residues in bromodomains can form van der Waals interactions with residues from the WPF shelf and the hydrophobic groups of inhibitors [115]. Specifically, the small hydrophobic gatekeeper residue Val419 in PfBDP1-BRD is conserved with family II BET-BRD members, which possess the ability to select multiply acetylated histones [116]. Phillips’s research also unveiled that K5ac is coordinated by at least four hydrophobic contacts, one of which involves the ATAD2B BRD gatekeeper residue I1048. The X-ray structures indicate that the size of the gatekeeper side chain, such as Val1174 in CREBBP, Ile1950 in BAZ2B, and Phe714 in BRPF1b bromodomains, influences the orientation of the indole moiety [117]. The work of Spiliotopoulos et al. suggested that the different orientation of the pyrrole ring in complexes with BAZ2A and BAZ2B can be attributed to either the difference in gatekeeper residues or crystallographic constraints [107]. Cazzanelli and co-authors confirmed that various gatekeeper residues (Tyr814 in GCN5, Phe3013 in BPTF, and Val1879 in BAZ2A), along with the substitution of GCN5 Ala762 or BPTF Ala2961 with BAZ2A Val1827, cause the 5-amino-4-bromo-2-methyl-3-pyridazinone headgroup to tilt differently [19]. Based on these analyses, gatekeeper residues across BRD members indeed influence the binding and orientations of inhibitors. Hence, gatekeeper residues have the potential to enhance selectivity for a single bromodomain target or a small subset of bromodomains in future drug design targeting the BRD family.

### 2.5. Discoveries of New BRD Targets

**Figure 5:**
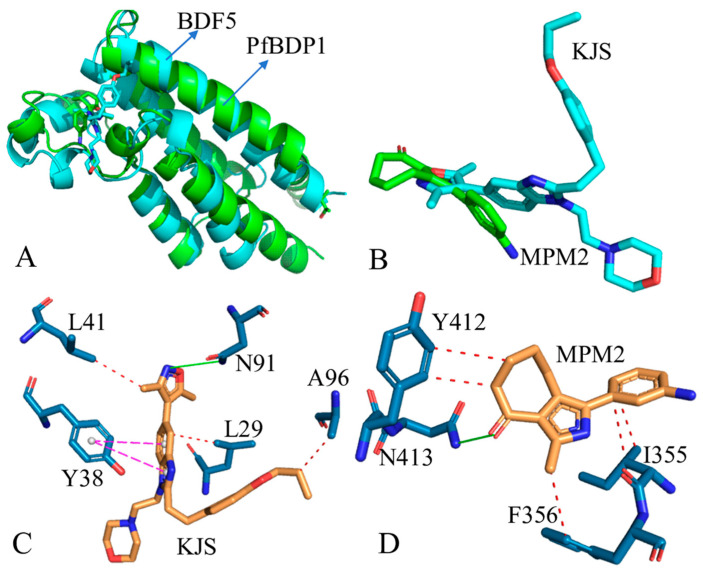
Structures of BDF5 and PfBDP1, together with their inhibitors: (**A**) structure superimposition of BDF5 and PfBDP1, (**B**) poses of inhibitors KJS and MPM2 in binding pocket of BDF5 and PfBDP1, (**C**) hotspots of KJS-BDF5 binding, and (**D**) hotspots of MPM2-PfBDP1 binding. In this figure, proteins are shown in cartoon modes, while inhibitors and key residues are depicted in stick modes. In this figure, the red dot lines indicate hydrophobic interactions, the green solid lines represent hydrogen bonding interactions and the purple dot lines describe the π-π interactions.

Efforts to identify novel BRD targets and develop corresponding inhibitors remain ongoing, with several BRD targets having been discovered to date [118]. Bromodomain-containing protein 8 (BRD8) has been identified as a subunit of the NuA4/TIP60 histone acetyltransferase complex [119,120,121,122]. Additionally, BRD8 has been implicated in cancer development through both NuA4/TIP60 complex-dependent and -independent mechanisms. Leishmania donovani bromodomain factor 5 (LdBDF5) represents a promising target for antileishmanial drug discovery. BDF5 contains a tandem pair of bromodomains (BD5.1 and BD5.2) at its N-terminus [123] and serves as an essential regulator of transcription in Leishmania [124]. Lin et al. determined the crystal structure of BDF5 in complex with the ligand KJS (PDB: 6NEZ), providing valuable structural insights for the rational design of BDF5-targeted inhibitors. Furthermore, SGC-CBP30 has demonstrated activity against Leishmania promastigotes in cell viability assays [123]. More recently, Plasmodium falciparum bromodomain protein 1 (PfBDP1) has emerged as a key target for drug design within the BRD family [125], with inhibitors MPM2 and RMM23 exhibiting promising inhibitory activity against this target [125]. Figure 5A presents a structural superposition of BDF5 and PfBDP1 bound to inhibitors KJS and MPM2, respectively, with the chemical structures of MPM2 and KJS illustrated in Figure 5B. In the BDF5–KJS complex, binding primarily involves residues L29, Y38, L41, N91, and A96 (Figure 5C). Specifically, the alkyl side chain of L29 participates in CH–π interactions, while the alkyl groups of L41 and A96 engage in CH–CH interactions with KJS (Figure 5C). The phenyl ring of Y38 contributes π–π stacking interactions with the hydrophobic rings of KJS, and N91 forms a hydrogen bond with the ligand (Figure 5C). Regarding the PfBDP1–MPM2 interaction, residues I355, F356, Y412, and N413 constitute the critical binding hotspots (Figure 5D). The alkyl chain of I355 and the phenyl ring of F356 establish CH–π interactions with the phenyl and alkyl moieties of MPM2, respectively (Figure 5D). Meanwhile, the hydrophobic ring of Y412 generates π–π stacking with MPM2, and N413 participates in hydrogen bonding with the inhibitor (Figure 5D). These identified residues may serve as important determinants in the design of selective inhibitors targeting the BRD family.

### 2.6. Investigation of BRD Inhibitors Aided by Computer Simulations

Predicting the 3D structure of bromodomains and the binding mode of inhibitors to BRD members is achievable through AlphaFold, molecular docking, and MD simulation. When experimental methods fall short in swiftly and precisely parsing the dynamic changes of BRD structures, computational simulation serves as an effective supplement, laying the foundation for subsequent drug design. These tools can pinpoint potential binding sites in bromodomains, including their unique conservative structures and specific amino acid residues. For example, residues such as Asn140, Tyr97, and Ile146 in bromodomains may play a crucial role in the binding of small-molecule inhibitors, thereby offering theoretical support for drug design targeting the BRD family. To explore the selective binding of inhibitors to various BRD proteins and clarify the molecular mechanism of selectivity, MD simulations, binding free energy calculations, and principal component analysis have been employed [16,23,32,126]. The integration of MD simulations with the Markov model has unveiled the allosteric regulation and binding mechanism of BRD4 and BRD9 [127,128], providing rational conformation states for drug screens. Molecular docking and MD simulations have been combined to investigate the binding selectivity of inhibitors and discover potent and selective BRD4 inhibitors capable of blocking TLR3-induced acute airway inflammation [129,130]. Additionally, QM/MM calculations have been applied to study the binding mechanism of inhibitors to BRDs and offer accurate quantum chemical information for drug design targeting BRDs [66].

A novel approach known as the residue-based reaction map method, which integrates molecular dynamics (MD) simulations and machine-learning techniques [79,80,81,82,131], has been developed. This method can compute a variety of properties of protein residues from conformations extracted in MD trajectories, whether in the presence or absence of ligand binding. The computed properties are then input into random forest and deep neural network models, which can predict binding states and assess each residue’s contribution to conformational changes following allosteric modulator binding [131,132]. This innovative strategy provides new descriptors and identifies key sites for drug design. For instance, Wang and colleagues combined MD simulations and deep learning to explore the binding selectivity of inhibitors to BRD4 over BRD9, offering valuable molecular-level insights for drug development [89]. In their work, three inhibitors, H1B, JQ1, and TUV, are involved in their investigation of inhibitor selectivity. Meanwhile, Jiang and co-workers employed machine-learning algorithms trained on prior structural and activity knowledge to predict the probability that a compound is a BRD4 inhibitor based on its binding pattern with BRD4 [85]. For their investigation, inhibitors of BRDs in clinical trials, including RVX-208, OTX015, IBET-762, CPI0610, and ABBV075, were used to guide drug design via machine learning, and a series of small-molecule compounds were designed. Based on the aforementioned works, machine learning can be performed on data from the experiments, MD simulations, and molecular docking to quickly screen potential inhibitors toward the BRD family. With the swift advancement of computer and artificial intelligence technologies, the methods that integrate machine learning and conformational sampling are expected to play an increasingly significant role in the development of BRD inhibitors. This development holds the potential to facilitate the creation of clinically useful BRD inhibitors in the future.

## 3. Conclusions

The discovery of the bromodomain as a specialized acetyl-lysine binding module laid a solid foundation for its emergence as a promising drug target. Various studies indicated that bromodomains are druggable targets, paving the way for the development of small molecules capable of displacing acetylated peptides from the conserved binding pocket of a bromodomain. This review aims to provide a concise explanation of the common structural topology among all BRD family members and summarize some inhibitors targeting this structural topology. We describe selective inhibitors for BRD2, BRD3, BRD4, and BRDT, as well as their interaction modes. Additionally, we cover selective inhibitors for BRD9 and BRD7, and those for BAZ2A and BAZ2B. The impact of gatekeeper residues on the binding of inhibitors to BRDs is also clarified, which should be given special attention in future drug development. Furthermore, computer simulation technologies, such as molecular dynamics (MD) simulations, QM/MM calculations, and machine learning, play significant roles in the study of BRD inhibitors. These technologies can serve as efficient tools for designing BRD inhibitors.

## 4. Future Perspective

Bromodomain inhibitors, especially BET bromodomain inhibitors, have shown great potential in the treatment of various diseases, particularly in cancer therapy. Numerous preclinical studies have demonstrated that bromodomain inhibitors can downregulate key genes promoting cell-cycle progression, survival, and inflammation, leading to growth inhibition in preclinical models of solid tumors and hematologic malignancies. In vitro and in vivo studies have shown that inhibition of BRD binding can suppress tumor growth associated with dysregulation of transcription factors such as MYC. Some small-molecule inhibitors of bromodomains have exhibited inhibitory effects on cancer cell proliferation and reduced the sizes of 3D cancer cell spheroids. For instance, certain compounds have induced cell-cycle arrest in the G0/G1 phase in breast cancer cells and significantly decreased the growth of T47D cells. In clinical trials, although none of the BRD inhibitors has yet received regulatory approval, some encouraging emerging data have been reported. Multiple small-molecule BET bromodomain inhibitors are undergoing clinical trials, and their clinical pharmacokinetic and safety profiles have been preliminarily evaluated.

In AI, machine-learning and deep-learning algorithms can swiftly screen a vast number of compound molecules. By analyzing and learning from the structures and properties of compounds, they establish predictive models to rapidly identify compounds with potential bromodomain inhibitory activity. This significantly reduces screening time and enhances research and development efficiency. Based on existing compound data, AI can predict molecular activity, selectivity, pharmacokinetics, and other properties. According to these predictions, it can then optimize molecular structures to quickly find inhibitor structures with better performance, thus reducing the time and cost of traditional trial- and-error experiments. AI can integrate data and knowledge from multiple fields, including chemistry, biology, and medicine. This breaks down disciplinary barriers, promoting communication and collaboration among researchers from different fields and accelerating the development of bromodomain inhibitors. In terms of human diseases, especially cancer, it is predicted that bromodomain inhibitors will have a promising future.

## Data Availability

Not applicable.
